# Temperature-Sensitive Materials for Oil and Gas Drilling Applications

**DOI:** 10.3390/molecules29071471

**Published:** 2024-03-26

**Authors:** Shuangchun Yang, Hao Wang, Yanchao Wang

**Affiliations:** Department of Petroleum and Natural Gas Engineering College, Liaoning Petrochemical University, No. 1, West Section of Dandong Road, Wanghua District, Fushun 113001, China; yangchun_bj@126.com (S.Y.); vangaaoe@126.com (H.W.)

**Keywords:** temperature-sensitive materials, oil and gas drilling, N-substituted acrylamide polymers, amphiphilic block copolymers, peptides

## Abstract

With the vigorous development of the petroleum industry, improving the efficiency of oil and gas exploitation has become an important issue. Temperature-sensitive materials show great potential for application in the development and production of oil and gas fields due to their unique temperature-responsive properties. This paper reviews the application of temperature-sensitive materials in oil and gas drilling and introduces the characteristics of three types of temperature-sensitive materials: N-substituted acrylamide polymers, amphiphilic block copolymers, and peptides. Because these materials can change their physical state at specific temperatures, this paper discusses in detail the role of various temperature-sensitive materials as plugging agent, thickener, oil displacing agent, flocculant, and tackifier in oil and gas field operations, as well as the mechanism of action and performance of temperature-sensitive materials in practical oil and gas drilling operations. As we have not yet seen relevant similar literature, this paper aims to discuss the innovative application of temperature-sensitive materials in the oil and gas drilling process, and at the same time points out the problems in the current research and applications as well as future development directions. Through analysis and comparison, we provide an efficient and environmentally friendly materials selection option for the petroleum industry in order to promote the progress and sustainable development of oil and gas extraction processes.

## 1. Introduction

In the 1960s, shape memory polymers were born. As early as 1941, Vernon [1] published the first medical patent related to “shape memory” materials. In the subsequent decades, various materials with “shape memory” characteristics were discovered. These materials can be categorized into categories such as electrically sensitive, temperature-sensitive, light-sensitive, and magnetically sensitive, depending on their response to different external conditions. Among these, temperature-sensitive materials have gained increasing attention from researchers due to their simple control methods and convenient preparation. They are currently widely used in various fields, including aviation, healthcare, electronics, construction, and the petroleum industry.

In the petroleum industry, whether in the drilling of oil and gas wells, the exploitation of oil fields, or the treatment of oil field sewage, experts and scholars at home and abroad have conducted extensive research and application. For example, Kamal et al. [2] introduced a new high-temperature, high-shear (HTHS) thermal-sensitive water-soluble polymer suitable for challenging conditions. These polymers contain temperature-sensitive monomers, and when the temperature exceeds the lower critical solution temperature (LCST), they form a physical network, leading to an increase in viscosity. This effectively addresses challenges in the extraction of oil from high-temperature, high-salinity reservoirs, and similar problems.

Furthermore, BASF [3] developed a temperature-sensitive polymer based on hydrophobically modified polyacrylamide to enhance oil recovery. The viscosity of this polymer can change with the change of temperature, and this heating and thickening behavior is reversible. During injection at surface temperatures, the fluid has a lower viscosity, allowing quick injection. Once it enters the reservoir, viscosity increases, thereby improving oil displacement efficiency.

In addition, Fan Guoqiang [4] synthesized graft polymers PAA-g-PNIPA and PAA-g-P(NIPA-CO-DMAA) using materials such as N-isopropylacrylamide (NIPA) and potassium persulfate (KPS). They prepared a temperature-sensitive thickener, PAA/PNIPA8, which exhibits high stability in high-temperature mud environments.

Furthermore, some researchers have explored topics such as the rheological properties of oil field working fluids and the modeling of the principles of temperature-sensitive polymer operation. The team led by Garmeh [5] developed two simulation methods to model the interaction of the properties of thermally active polymers (TAPs) with reservoir rocks. Both methods include temperature-triggered viscosification and adsorption/retention effects. Results showed that temperature-triggered polymers can increase oil recovery by viscosification and chemical adsorption/retention. Xie [6] synthesized a new thermo-sensitive copolymer (PANA) of acrylamide (AM), 2-acrylamido-2-methylpropane sulfonate (NaMAMS), and N-vinylcaprolactam (NVCL) under optimal conditions by free radical polymerization. Results from rheological tests illustrated that PANA showed certain thermo-thickening phenomena in the temperature range of 4–95 °C due to the thermo-associative behavior of thermo-responsive caprolactam (CL) moieties. Therefore, the relatively stable rheological properties of fluids with temperature changes could be achieved through the synergistic effect between the novel thermo-sensitive copolymer and bentonite particles.

This paper reviews the classification of temperature-sensitive materials and their application in oil and gas drilling. The uses of temperature-sensitive materials were classified and reviewed. It also looks forward to the future application of temperature-sensitive materials in the petroleum industry, aiming to provide a reference for relevant scholars.

## 2. Classification of Temperature-Sensitive Materials

Temperature-sensitive polymers refer to a class of polymers whose physical and chemical properties undergo drastic and discontinuous changes under the stimulation of environmental temperature [7,8]. According to the material’s composition and phase transition mechanism, temperature-sensitive high-polymer materials are generally classified into three major categories: N-substituted acrylamide polymers, amphiphilic block copolymers, and peptides. They all exhibit a relatively low lower critical solution temperature (LCST) [9]. Below the LCST, the polymer dissolves well in the solvent, forming a uniform solution. However, when the temperature exceeds the LCST, the polymer rapidly aggregates and precipitates from the solvent. The change of properties of temperature-sensitive materials is caused by specific groups in their molecular structure. These groups may exhibit strong interaction forces at low temperatures, allowing the material to maintain a stable structure and properties. As the temperature increases, the thermal motion of the molecule intensifies, and the original interaction force weakens, resulting in changes in the structure and properties of the material.

Among N-substituted acrylamide polymers, poly(N-isopropylacrylamide) (PNIPAAm) is the most widely used, mainly because it has a very small phase transition temperature range and an LCST (31 °C) close to human body temperature. PNIPAAm consists of both hydrophilic and hydrophobic amide groups and isopropyl groups. In 1968, Heskins [10] and Guillet studied the morphology and temperature-sensitive properties of PNIPAAm in aqueous solutions. Below the LCST, PNIPAAm can form a uniformly transparent solution with water, and the polymer chains exhibit an extended conformation. However, when the temperature exceeds the LCST, PNIPAAm quickly aggregates and precipitates from the water, causing the polymer chains to adopt a coiled configuration. These changes are reversible and discontinuous processes. In general, the polymer compounds obtained by copolymerization of NIPAAm with other monomers are also temperature-sensitive [11,12], and the temperature-sensitive effect depends on different monomers and their ratios.

Professor Wei Hua from Lanzhou University conducted research on the synthesis and performance of cyclic temperature-sensitive amphiphilic block copolymers [13]. First, they synthesized block copolymers using a two-step continuous RAFT polymerization method, followed by a one-pot amine exchange–Michael addition reaction to fully end-cap the RAFT groups. Finally, they cyclized the polymers using intramolecular click chemistry to obtain the desired cyclic block copolymers. The research indicated that cyclic temperature-sensitive amphiphilic block copolymers have a higher LCST compared with linear polymers.

Meng Zhu studied the preparation and properties of temperature-sensitive hydrogels composed of peptides [14]. The study focused on peptides composed of hydrophobic amino acids (leucine, isoleucine, and proline). They designed and synthesized a series of polyethylene glycol–peptide temperature-sensitive polymers and systematically investigated the effects of amino acid side-chain structures, chain lengths, copolymer compositions, and block order on the aggregate structure and temperature response behavior of the copolymers in aqueous solution. The research also explored the mechanisms of sol–gel transitions in different systems. The researchers constructed and studied temperature-sensitive hydrogels based on different polypeptide segments, revealing the relationship between the structure and function of polypeptide materials, which has important theoretical significance and practical application value. Table 1 summarizes the advantages and disadvantages of three types of temperature-sensitive polymer materials.

## 3. Temperature-Sensitive Materials for Oil and Gas Drilling Applications

### 3.1. Plugging Agent

In the processes of oil and gas drilling and production, lost circulation has always been a difficult technical problem to solve, and it is also one of the main sources of non-production time when drilling. Nowadays, lost circulation problems under various geological conditions have not been completely resolved [15]. As far back as the 19th century, attempts were made to prevent lost circulation by adding particulate materials to drilling fluids [16]. However, traditional lost circulation materials have limitations in adapting to different situations and achieving satisfactory sealing effects [17]. In order to solve this problem, the majority of scholars are committed to developing high-performance plugging materials to solve the problem of lost circulation.

In their research, Bao Dan et al. [18] presented advances in intelligent lost circulation materials based on temperature-sensitive shape-memory properties. They discussed the types and characteristics of mechanism-memory intelligent materials, and proposed suggestions and prospects for the preparation, evaluation methods, and field process schemes of temperature-sensitive shape-memory leak-stopping materials, which may promote the innovation of leak-stopping technology. Temperature-sensitive materials are of great interest due to their temperature-sensitive response, reversible changes, and high strength [19]. In addressing the issue of well leakage, the use of temperature-sensitive materials can exploit the interlayer temperature differences to trigger their characteristics, promoting rapid and efficient pressure sealing through properties such as expansion and bridging. This effect can be controlled by adjusting interlayer temperatures.

In recent years, temperature-sensitive materials have been widely used in the field of petroleum engineering [20,21] and have made remarkable progress, showing broad application potential.

In the oil and gas drilling process, the reservoir environment has a significant impact on production, especially in fractured formations where the loss pathways are extensive and the loss rate is high. Qiu and Xiao [22,23] found that this led to the plugging material being unable to form a structurally stable confined formation in the fracture, thereby increasing the difficulty of lost circulation operations in drilling fluids.

**Figure 1:**
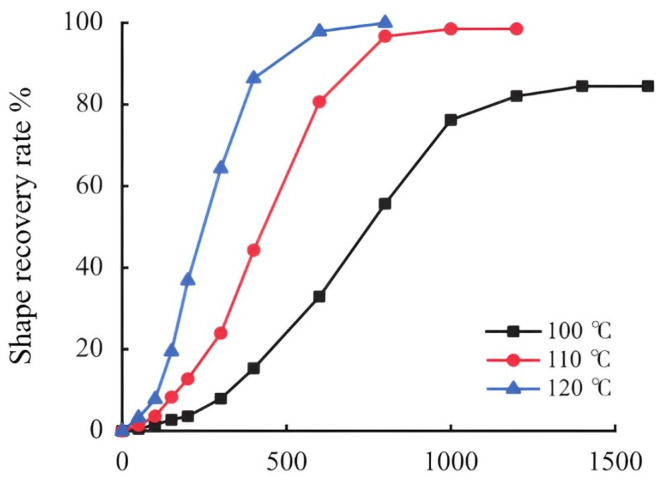
Shape-memory polymer recovery rate versus time [24].

Feng Jie et al. [24] prepared a shape-memory polymer plugging material with thermal activation characteristics based on the shape memory and recovery mechanism of shape-memory polymers. They cured epoxy resin monomers by adding amine accelerators and then used a hot press to crush, granulate, and sift the product, creating temperature-sensitive shape-memory plugging particles. These particles can be used to seal fractures of varying apertures, with a pressure-bearing capacity exceeding 9 MPa. Epoxy resin materials, due to their high strength and high density, are not easily compressed and undergo plastic deformation only at high temperatures, hence they cannot be used directly for preparing shape-memory leak-stopping agents [25]. Figure 1 shows the shape-memory polymer recovery rate versus time.

**Figure 2:**
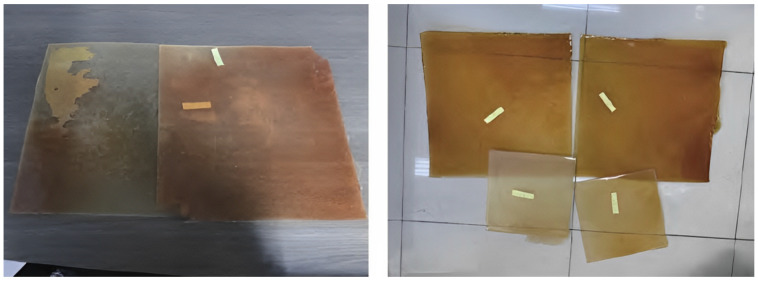
Shape-memory polymer thermoformed sheet [29].

Liu Zhaonian et al. [26] used a curing agent under certain temperature conditions that underwent an addition polymerization reaction with epoxy groups to generate a shape-memory polymer TS-LCM with a three-dimensional network structure. This temperature-sensitive shape-memory lost-circulation agent presents a two-dimensional structure at room temperature, and it expands into a three-dimensional structure when activated, displaying excellent high-temperature resistance, with an expansion rate exceeding 100%. It is not affected by the type of medium and has good compatibility with drilling fluid. Using the principles of epoxy–thiol click chemistry [27], Tang Longhao and colleagues [28] produced epoxy resin shape-memory polymers that can adjust their particle size, glass transition temperature, shape recovery rate, and shape fixation rate to meet the specific needs of downhole lost circulation. Wang Baotian and colleagues [29] developed temperature-sensitive shape-memory lost circulation materials by using low-monomer resin monomers and different high-temperature crosslinking agents, as shown in Figure 2.

After aging, this material can self-adapt to crack bridging and plugging, improving the retention ability and pressure resistance of the plugging fluid.

Currently, a variety of plugging systems have been developed, among which termperature-sensitive (reversible) gels are a type of polymer with non-toxicity and strong antibacterial properties [30,31,32]. They exhibit unique thermo-reversibility, forming and dissolving gels multiple times with temperature changes. The gel temperature and viscosity can be altered by adding electrolytes or non-electrolytes [33]. When the environmental temperature is below the gelation temperature, the already-gelled semi-solid temperature-sensitive gel can revert to a low-viscosity liquid. This feature ensures safety during construction, as even if the gel forms in the wellbore or screens, or even if it completely blocks the reservoir, it can be unclogged by injecting cold water, thereby avoiding well abandonment. Temperature-sensitive gel plugging technology has yielded significant anti-leakage, sealing, and enhanced oil recovery effects in some oil fields [34,35]. Li et al. [36] proposed a temperature-sensitive granular gel with an upper critical solution temperature (UCST). This gel presents a microscopic network structure. With the increase of temperature, the gel undergoes a change of expansion–contraction–expansion. Using its characteristics, the function of efficient plugging can be realized.

Mei Wei and colleagues [37] investigated the plugging effect of temperature-sensitive reversible gels under different injection rates and times. They found that at 80 °C, the plugging effect of the temperature-sensitive reversible gels was most effective, and displacement efficiency increased with higher injection volumes. Furthermore, when the gel injection volume was close to the channel volume coefficient, ideal plugging effects were achieved. Temperature-sensitive gels can effectively plug high-permeability zones in the reservoir, thereby mitigating gas leakage issues.

Zhang Wei [38] studied a temperature-sensitive gel plugging agent through static and dynamic laboratory experiments and optimized a reversible temperature-sensitive gel and foam system suitable for offshore heavy oil reservoirs. They optimized the selection of suitable reversible temperature-sensitive gel and foam systems for offshore heavy oil reservoirs. Using different construction methods, such as pre-segment plug injection and in-situ plug injection during heating, they carried out composite plugging. The experimental results showed that after using temperature-sensitive gel plugging technology, temperature-sensitive gels effectively sealed the flow channels formed during two heat cycles and increased the heating injection pressure. No gas leakage occurred throughout the heating process, and it had no impact on production in the surrounding wells.

Feng Xiang and colleagues [39] studied the application of temperature-sensitive gel chemical plugging technology for suppressing gas channeling. They analyzed the mechanism of temperature-sensitive gel-assisted multi-thermal fluid displacement and optimized it through numerical simulation methods, providing technical support for the prevention and control of gas channeling in offshore thermal recovery wells. Additionally, Wang Cheng [40] investigated the plugging performance of temperature-sensitive gels primarily composed of cellulose ether and found that the temperature-sensitive gel could form a solid at certain temperatures, providing excellent plugging effects in high-permeability reservoirs. Su Yi [41] successfully prevented gas channeling issues and improved the production efficiency of thermal recovery wells by selecting temperature-sensitive reversible gels and enhancers and applying appropriate construction methods in an oilfield where gas channeling was a severe problem during the heating injection period.

**Figure 3:**
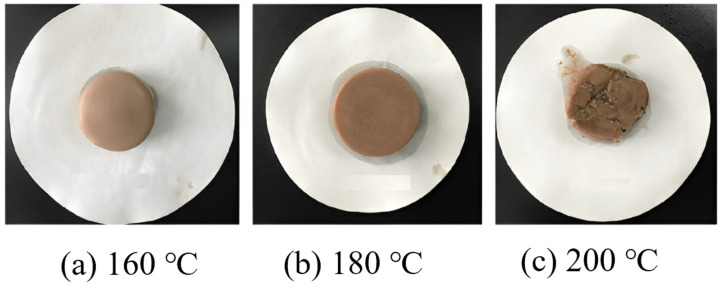
Formation of the fabricated temperature-sensitive gel at different temperatures [42].

Zhang Kun’s research [42] focused on temperature-sensitive gel lost-circulation agents. It addressed the issues of poor controllability of gel formation and low structural strength at high temperatures. They developed a temperature-sensitive gel lost-circulation agent that can form a robust gel between 80 °C and 180 °C and exhibits a broad spectrum of temperature-sensitive effects. Figure 3 illustrates the formation of the temperature-sensitive gel at different temperatures. This gel plugging agent solves the problem of high-temperature gelation of large-particle solid phase, and can be used in combination with other granular and fibrous plugging agents to improve the pressure bearing capacity of the formation. Xie Zhiqin [43] preferred refined cotton fiber as the main raw material to prepare a temperature-sensitive thermoreversible gel system and investigated the effects of different additives on the system, and the results showed that inorganic salts could reduce the gel-point temperature of the system. This is mainly because inorganic salts affect the interaction force between polymer chains by changing the ionic strength and charge shielding effect of the solution, so they can form a stable gel structure at a lower temperature.

The approach to designing a temperature-sensitive cement slurry system for plugging leaks is as follows: at room temperature, temperature-sensitive shape-memory materials with small particles are used for easy mixing and pumping. When it enters the wellbore and is heated to the material’s temperature-sensitive point, the material’s volume expands, forming large frameworks or long fibers and providing bridging effects to cooperate with cement slurries for plugging [44,45,46,47,48,49,50,51]. Based on a profound understanding of the structure of organic crosslinked gels, research teams led by Liu Wei [52] used polyethyleneimine (PEI) as a crosslinking agent to successfully prepare an elastic liquid plug EGL-1. This plug significantly enhanced the reservoir’s pressure-bearing capacity, effectively preventing downhole fluid loss and reducing reservoir damage.

Overall, temperature-sensitive plugging materials possess numerous excellent properties, holding significant practical value and development potential for complex geological formations. However, temperature-sensitive materials are still in the development stage, and future research should focus on the adaptability of products and comprehensive performance analysis. This should be done in conjunction with optimizing technical solutions based on the environmental characteristics of the construction site, thereby promoting the application of temperature-sensitive lost-circulation materials in actual engineering.

### 3.2. Thickener

**Figure 4:**
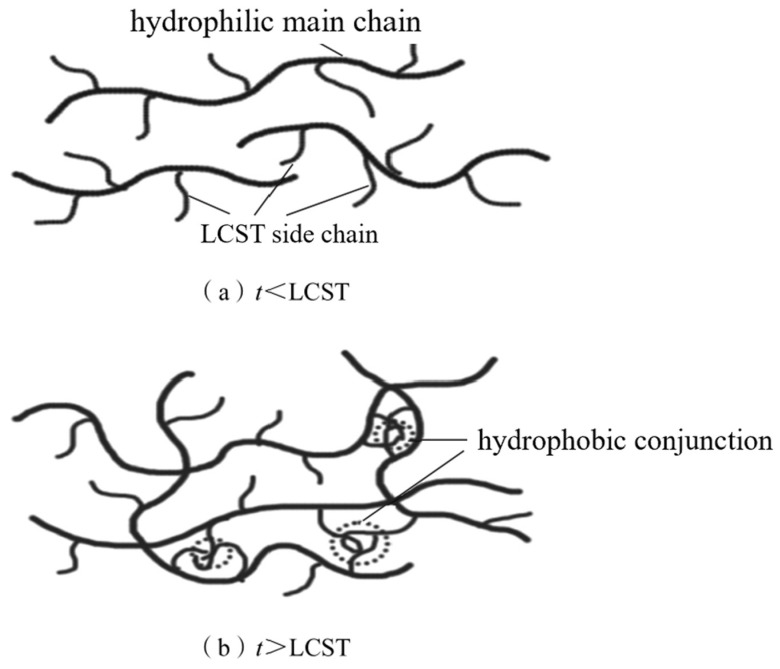
Conjugation mechanism [62].

**Figure 5:**
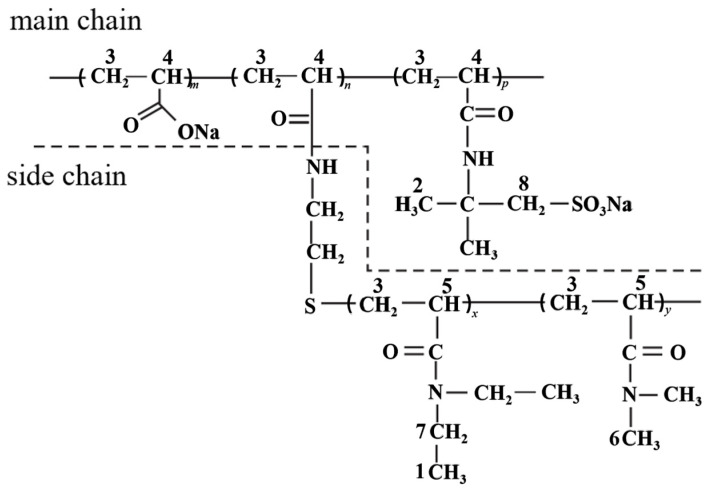
Structure of temperature-sensitive thickening graft polymer [62].

With the gradual maturation of technology, oil and gas exploration continues to explore deeper subsurface layers. The number of deep and ultra-deep wells continues to increase, leading to complex wellbore structures, and the circulating temperature at the bottom of the wells gradually rises, thereby increasing the complexity of well cementation [53,54]. To meet these high-difficulty construction requirements, it is often necessary to add some additives to adjust the performance of drilling mud. However, while these additives can improve performance, they may also bring some adverse effects. For instance, retarders may exhibit strong dispersibility at high temperatures, which can lead to muddy dilution and reduced stability and ultimately results in an uneven density of the well cement slurry, making it ineffective in isolating oil, gas, and water layers. To address this issue, thickeners are usually added [55,56,57]. Commonly used thickeners include xanthan gum and its modified derivatives [58]. Temperature-sensitive thickeners typically involve molecular structural forces, intermolecular forces, and self-assembly behavior. When the temperature increases, the hydrogen bonds break and the interaction force is weakened, thus affecting the viscosity of the materials. Some temperature-sensitive thickeners can form special structures through self-assembly behavior. These structures are entangled with each other in the solution to form a network structure to increase viscosity. However, such materials, despite their excellent salt resistance and high temperature resistance, exhibit significant thickening problems at low temperatures, making them unsuitable for practical engineering applications. In response to this issue, in the 1990s, French scientist Hourdet proposed a new idea of “thermoreactive thickening polymers” [59]. In recent years, researchers have applied the rheological properties of temperature-sensitive polymers to solve difficult problems in various operations of oil fields, such as improving the temperature resistance and salt tolerance of tertiary oil recovery polymers [60], enhancing the high-temperature stability of cement slurry, and adjusting the rheological properties of drilling fluids, all of which have achieved good laboratory research results [61]. To address the problem, Guo Jintang and his colleagues [62] designed and synthesized an additive with a high-temperature thickening effect, which not only compensates for the dilution of polymer-based cement additives at high temperatures but also maintains stability at high temperatures. This temperature-sensitive polymer consists of a main chain made up of the hydrophilic polymer P(AA-co-AMPS), and side chains of thermosensitive polymers P(DEAA-co-DMAA) with a lower critical solution temperature (LCST). It is prepared by grafting crosslinking to form water-soluble grafted polymers with controllable structures. Crosslinking between these molecules helps to form a physically networked structure, thereby significantly enhancing the thickening performance [63,64,65,66,67,68]. The principle is shown in the figure below. The introduction of AMPS is not only favorable for grafting reactions but can also enhance the polymer’s high-temperature and salt resistance [69]. Figure 4 and Figure 5 respectively illustrate the conjugation mechanism and structure of temperature-sensitive thickening polymers.

In order to solve the problem that temperature-sensitive thickeners have insufficient salt and temperature resistance and are difficult to adapt to high-temperature reservoirs, Xu and his team [70] synthesized a heat-sensitive copolymer based on the monomer N,N-diethylacrylamide (DEAM) that exhibits heat-thickening characteristics only at relatively high concentrations. Chen Luxin and others [71] used self-made temperature-sensitive monomers (PADA), 2-acrylamido-2-methylpropanesulfonic acid (AMPS), N,N-methylenebisacrylamide (MBA), and modified nano-SiO_2_ particles (N-np) to prepare a temperature-sensitive polymer/nano-SiO_2_ composite material, N-AMPA. This material exhibits heat thickening at low concentrations and excellent high-temperature resistance, salt resistance, and shear resistance, with a maximum thickening rate of 94%. N-AMPA solution retained 68% viscosity after aging at 200 °C; in a 20% NaCl brine solution, the viscosity retention rate reached 63%; at a shear rate of 1021 s^−1^, the solution viscosity reached 50 mPa·s. Compared with the temperature-sensitive polymer AMPA, N-AMPA shows outstanding high-temperature, salt, and shear resistance.

In drilling fluid systems, thickeners are essential components for reducing the rate of hydrogen ion transfer and controlling filtration loss [72]. Acid thickening agents are common choices, and polyacrylamide-based thickeners are commonly used. However, their main problem is that the viscosity of the solution drops sharply with the increase of salinity and temperature [73]. Some common temperature-sensitive polymers include poly(N-isopropylacrylamide) (PNIPAAm) and polyethylene oxide (PEO), both of which have linear structures [74,75,76]. Dai Shanshan and her team [77] adopted the free radical copolymerization of allyl polyoxyethylene ether (APEG) with long-chain temperature-sensitive groups and acrylamide (AM) to prepare a new type of binary temperature-sensitive acid fluid thickener P(AM-APEG), and tested its temperature-sensitive performance in comparison with PAM that did not include temperature-sensitive groups. The results show that the synthesized P(AM-APEG) exhibited relatively minor viscosity changes at different temperatures, with viscosity slowly decreasing as the temperature rose. Based on these experiments, their team also conducted free radical copolymerization of acrylamide (AM), dimethylacrylamide-oxyethyl trimethyl ammonium chloride (DMC), and allyl polyoxyethylene ether with long chain temperature-sensitive groups (APEG), preparing a new type of temperature-sensitive acid fluid thickener P(AM-DMC-APEG). Temperature-sensitive thickening agents can be used to control the rheological properties and filtration loss of drilling fluids, sealing materials, and third-stage oil recovery, and are expected to improve high-temperature resistance and construction performance.

Most temperature-sensitive thickening materials are applied in the third-stage exploitation process of oil fields. They provide thickening effects while increasing tolerance to high-temperature environments. Furthermore, the temperature adjustability of some temperature-sensitive thickening materials allows them to adapt to various complex geological conditions. However, the high-temperature resistance of most temperature-sensitive thickeners is currently limited and makes them challenging to apply in high-temperature reservoirs. Therefore, it is recommended to continue research on the high-temperature resistance of temperature-sensitive thickeners in the future.

### 3.3. Oil-Displacing Agent

The mechanism of polyacrylamide and its derivatives as oil displacement agents includes two main aspects: firstly, increasing the solution’s viscosity through the long-chain structure of molecules and, secondly, relying on the adsorption, mechanical trapping, and hydraulic trapping of oil displacement agents in the formation to reduce the permeability of the water phase. The adsorption is due to the combination of polymer macromolecules and the surface of the porous medium through hydrogen bonds and electrostatic forces, thereby forming an adsorption layer on the surface of the medium. This adsorption phenomenon results in the loss of flow ability of the polymer molecules, which reduces the permeability of the fluid in the pore medium. Mechanical trapping occurs when the temperature-sensitive oil displacement agent flows through the porous media of the formation; due to the complex pore structure, some polymer molecules may not be able to smoothly pass through the smaller pore throats and become physically trapped or stuck in the pores. Hydraulic trapping refers to the retention of polymer molecules at specific locations in the porous media due to the influence of hydraulic factors such as flow rate and pressure during the flow of polymer solutions. These two actions work together to reduce the ratio of water to oil phase mobility, increase the sweep efficiency, and, thus, enhance oil displacement effectiveness [78,79,80]. However, under high-temperature and high-salinity reservoir conditions, the oil displacement efficiency of polymers is usually poor. To improve oil displacement effectiveness and reduce costs, the most commonly used method is to add small amounts of complexing agents and AMPS anti-salt monomers during polymer synthesis [81,82,83,84,85]. Their unique structures impart special performance characteristics to temperature-sensitive polymers.

**Figure 6:**
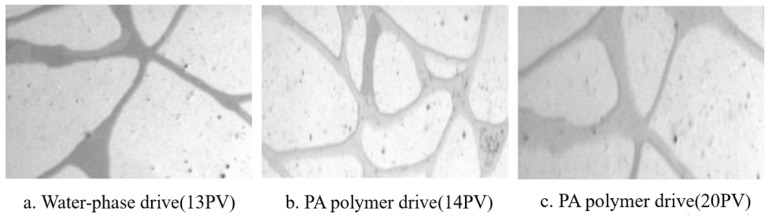
The microscopic oil displacement characteristics of PA solution [86].

For example, Ma Chao [86] studied the role of a temperature-sensitive polymer solution containing a benzene ring structure in oil displacement. He mainly explored the role of ternary copolymer temperature-sensitive polymers containing hydrophilic, hydrophobic, and temperature-sensitive monomers with benzene rings in the oil displacement process. Figure 6a shows an image of formation water flooding with a PA solution at the end. It illustrates that the residual oil partially adheres to the rock surface, and water flows out of the large channels. Figure 6b shows that when the PA solution is flooded out to 14 PV, the residual oil adhering to the rock surface peels off and emulsifies into an “oil band,” moving to the end of the pore. The hydrophilic base of the temperature-sensitive polymer’s polymer chain adsorbs onto the rock, exposing the hydrophobic portion, leading to wettability inversion, reducing the adhesion of the oil phase, and causing the oil to detach from the rock. The detached “small particle oil droplets” are then gathered by the temperature-sensitive polymer solution to form a “moving oil band” that migrates through the pore. Finally, Figure 6c illustrates that when the PA solution is flooded to 20 PV, the formed “oil band” has been removed from the pore.

**Figure 7:**
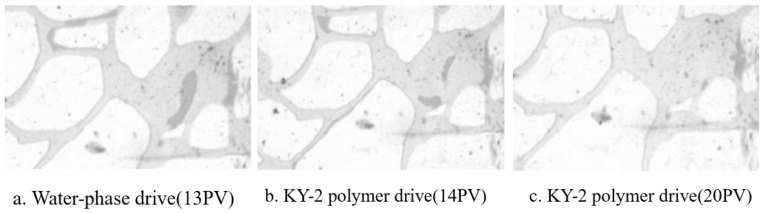
The microscopic oil displacement characteristics of polymer (KY2) solution [86].

Figure 7a shows an image from the end of the formation water flooding (13 PV). It indicates that, at the end of water flooding, some residual oil adhered to the rock surface (oil is in the upper left corner), and the oil in the middle was in large channels, with the aqueous solution not having taken away the oil from the large channels. In contrast, Figure 7b depicts that when the KY-2 solution was driven to 14 PV, the polymer solution did not completely strip the oil residue from the rock surface, only moving away some of the oil beads in the larger pores. This is because KY-2 failed to effectively reduce the oil–water interfacial tension, lacking the characteristic of temperature-sensitive polymer solutions to reduce the adhesion between oil and rock. Lastly, Figure 7c shows that at 20 PV, some small particle oil droplets were still not removed from the pore, indicating that KY-2 lacked the emulsification ability to significantly aggregate “small particle oil droplets” into an “oil band”.

The wettability of the rock surface determines whether the capillary force is the driving force or the resistance of oil displacement. If the wettability of the rock surface is hydrophilic, the capillary force is the driving force of oil displacement. If the contrary is the case, the capillary force is the oil displacement resistance. Therefore, the wettability of the oil-wet rock surface is changed to hydrophilicity, which is beneficial to the expulsion of crude oil [87]. Ma Jinhai and his team [88] studied a reverse temperature-sensitive gel oil displacement agent. First, they prepared N-allyl isobutylketone diamine by reacting allyl chloride with isobutylketone diamine. Then, they reacted it with acryloyl chloride to prepare monomers containing N-allyl, N-isobutyl, and N-isobutylketone diamine. Next, they dissolved this monomer with N-isopropyl acrylamide and acrylamide in a 1:3:6 ratio in distilled water. Using potassium persulfate as an initiator, they initiated copolymerization at 70 °C, resulting in a reverse temperature-sensitive active polymer gel. This gel’s aqueous solution had strong oil displacement capabilities and a reliable preparation process. Hendraningrat et al. [89] carried out experimental tests with hydrophilic nano-SiO_2_. When the concentration of nano-SiO_2_ in the solution increased, the contact angle of the liquid phase gradually decreased, and the wettability of the rock was reversed. Mohajeri et al. [90] developed nano-ZrO_2_ particles by the sol method and mixed them with anionic surfactant and cationic surfactant, respectively. The results showed that when the mass fraction of nano-ZrO_2_ in the solution was 0.01%, the contact angle of the oil phase decreased from 100° to 30° and 15°, respectively, and the wettability obviously changed. In the field of polymer grafting modification of nano-materials, on the basis of Janus nano-SiO_2_, Shi et al. [91]. obtained Janus particles with targeted migration function by coupling-agent modification and synthesized amphiphilic temperature-sensitive polymer microcapsules by atom transfer radical polymerization. Based on the temperature-response characteristics of the capsule wall, the intelligent targeted oil displacement of Janus nanoparticles was realized. The core flooding experiment showed that the water flooding recovery was reduced by 19.77% when the permeability ratio increased from 2.05 to 21.57. The recovery rate of the Janus intelligent microcapsule dispersion system was increased by 9.59%. Chen et al. [92] prepared temperature-sensitive Fe_3_O_4_ nanoparticles (TSIO). TSIO-based nanofluids can change the wettability of rocks and reduce the oil/water interfacial tension, thereby significantly improving oil recovery. Compared with water flooding, the oil recovery of TSIO-based nanofluid flooding increased by 12% at room temperature to 74%. The recovery efficiency reached 84% at 50 °C.

Although some research results have been achieved in temperature-sensitive oil displacement materials, they still have weaknesses in terms of pressure tolerance and stability [93,94,95,96,97,98,99]. Therefore, it is recommended to continue efforts in material selection and optimization, as well as further organization of material compositions to improve the oil displacement effectiveness of temperature-sensitive materials. The development of new temperature-sensitive materials, such as temperature-sensitive microcapsules and temperature-sensitive nanogels, is also worth exploring further.

### 3.4. Flocculant

Deng et al. [100] improved temperature-sensitive polymers (PNIPAM) by using cationic copolymer monomers, further enhancing the polymers’ dewatering characteristics and promoting the development of temperature-sensitive polymer applications. Sakohara and colleagues [101] used cationic and anionic modified PNIPAAm copolymers to flocculate TiO_2_, while proposing a new concept of sedimentation/compaction for flocculation. Researchers believed that by altering the suspension, the molecular chain of the polymer PNIPAAm can be transformed from hydrophilic to hydrophobic, thus achieving the flocculation effect of sedimentation/compaction. Franks and others [102] utilized temperature-sensitive polymers as secondary precipitation consolidants to further increase the dewatering rate of flocculants. Yi and his team [103] used cationic-modified NIPAAm copolymers containing 2.2 mol% diethylaminoethyl methacrylate (DEAA) in colloidal suspensions of 266 nm-sized TiO_2_ clusters to test their flocculation effects. The researchers found that flocculation occurred more rapidly at temperatures between 23 °C and 42 °C. Mirian and colleagues [104] reported the flocculation effects of using N-vinyl caprolactam (PNVCL) instead of PNIPAAM as a temperature-sensitive polymer. They studied flocculation with different pH values and temperature conditions and found that the flocculation sediment was denser and had lower water content when chitosan and a chitosan–PNVCL mixture were present. However, so far, research on temperature-sensitive polymeric flocculants has mainly focused on suspensions, with very little study on the mechanism in the actual treatment of waste fluid after oil and gas drilling and extraction. Sakohara [105] conducted research on the flocculation of industrial wastewater using temperature-sensitive polymers, indicating that the flocculation process of temperature-sensitive polymers is influenced by salt, acidity/alkalinity, and possibly other substances. The primary influencing factors are the polymer’s molecular weight, molecular structure, suspension pH, and temperature. In addition, the polymer dosage, the addition method, and the heating method for the suspension all affect the flocculation treatment. Yanan Zhang [106] conducted research on the synthesis of temperature-sensitive polymers and their flocculation effects in waste drilling fluid. They synthesized cationic temperature-sensitive polymer PAA by using temperature-sensitive monomers N,N-diethylacrylamide (DEAM), hydrophobic monomers (PB), hydrophilic monomer acrylamide (AM), and cationic monomers (PC) as reactive monomers through the redox micelle polymerization method. The research results showed that the highest transmittance of the clarified solution was achieved at a flocculant concentration of 200 mg/L, a flocculation temperature of 45 °C, and a pH control of 10. Sarang [107] investigated the effect of multifunctional poly (N-isopropyl acrylamide/acrylic acid/N-tert-butylacrylamide) [p(NIPAM-AA-NTBA)] ternary polymer on the sedimentation of kaolin clay—a major fraction of oil sands tailings. The ternary copolymer, used as a flocculant, exhibited thermo-sensitivity. As the ternary polymer is temperature-sensitive, it undergoes extension to a coil-like conformation. Settling tests at 50 °C resulted in significantly higher settling rates compared with that at room temperature. Wu Yayue [108] conducted comparative experiments on the flocculation of kaolin suspensions with Fe(OH)_3_-p[NIPAM-co-DMAPMA], p[NIPAM-DMAPMA], and p[NIPAM-DMAPMA]+Fe(OH)_3_. The results showed that adding the polymer at room temperature and flocculating under room temperature sedimentation (RMHS) conditions yielded the best flocculation effect. Flocculating with the polymer at high temperature and flocculating under high-temperature sedimentation (HMHS) conditions yielded the poorest flocculation effect. The treatment of waste drilling fluid is especially important, particularly in the current context of environmental protection. The authors suggest strengthening the research and application of temperature-sensitive flocculants to ensure good treatment results in various scenarios.

Currently, temperature-sensitive flocculants are often used in the treatment of waste drilling fluids. The emergence of temperature-sensitive flocculants has effectively overcome problems such as poor flocculation effect and weak adaptability of conventional flocculants. The removal of oil stains and other solid impurities from wastewater has been achieved by introducing different hydrophilic and hydrophobic groups, thereby purifying oilfield wastewater. However, the variety of temperature-sensitive flocculants is currently limited, and research directions are relatively narrow. The authors of this paper suggest that there should be a strengthening of research on the diversification of temperature-sensitive flocculant types.

### 3.5. Tackifier

**Figure 8:**
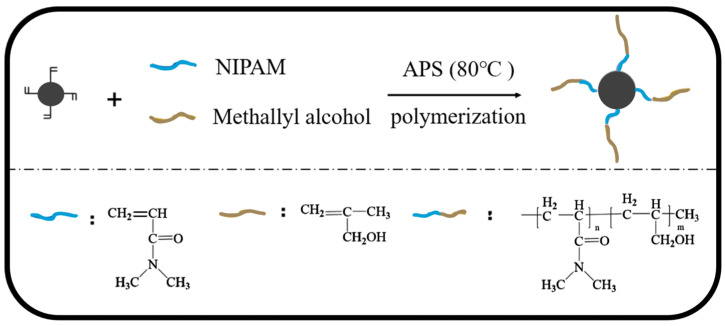
Preparation of temperature-sensitive nano-SiO_2_ [112].

In recent years, drilling fluid additives have undergone rapid development. Among these, types of tackifiers are increasing day by day. Common tackifiers include fatty acids, modified lignin, and oil-soluble resins, among others [109]. Common thermosensitive monomers include poly(ethylene oxide)-poly(propylene oxide) (PEO-PPO), poly(N-isopropyl acrylamide) (PNI-PAM), and macromonomer copolymers based on acrylamide dipropionate (MPAD) [110]. Temperature-sensitive polymers exhibit properties similar to conventional polymer solutions at room temperature. However, when the temperature exceeds the LCST (lower critical solution temperature), the thermosensitive groups physically intertwine with each other to form hydrophobic microdomains, resulting in a significant increase in solution viscosity [111]. Guo Miao [112], aiming to enhance the suspension stability and thickening properties of bentonite and maintain excellent rheological performance at high temperatures, successfully developed a temperature-sensitive nanosilica hybrid material as a tackifier for oil-based drilling fluids. Figure 8 demonstrates the preparation method of temperature-sensitive nanosilica. This tackifier exhibited outstanding performance at 150 °C, improving the stability and thickening properties of drilling fluids.

Facing the challenges of low-temperature, high-pressure environments, Gu Tiantian [113] prepared a temperature-sensitive polymer using poly-N-vinylcaprolactam (PVCL), with hydrophobic carbon chains and cyclic amides as basic units. This polymer forms a spatial structure and shows significant effects in practice. It halves the viscosity variation of drilling fluid, especially showing stability and regulation ability at high temperatureS.

Li Yanjun and colleagues [114] addressed the inability of conventional deepwater drilling fluids to cope with deepwater high-temperature, high-pressure subsurface environments by developing a temperature-sensitive tackifier cutting agent and conducting performance evaluations. The results showed that this temperature-sensitive tackifier maintained the rheological properties of drilling fluids and exhibited excellent high-temperature and low-temperature resistance.

Chen et al. [71]. prepared a temperature-sensitive polymer/nano-SiO_2_ composite N-AMPA by in situ polymerization using self-made temperature-sensitive monomer (PADA) and modified nano-SiO_2_ as the main body. The composite material had obvious temperature-sensitive thickening behavior at 65~180 °C, and the thickening rate reached 94%. N-AMPA can retain 68% viscosity retention rate after aging at 200 °C, and 63% in 20% NaCl aqueous solution.

Wang Mengmeng and colleagues [115] used acrylamide, sodium acrylate, and temperature-sensitive macromonomers as polymerization monomers to prepare a temperature-sensitive water-soluble polymer emulsion through a reverse-phase emulsion polymerization method. This tackifier quickly dissolved in drilling fluids and had a relatively long shelf life. At low concentrations, it maintained high viscosity and exhibited significant thermal thickening effects. These temperature-sensitive tackifier materials have outstanding performance and contribute to improving the rheological properties of drilling fluids. Future development efforts should focus on creating even more superior materials while reducing costs.

At present, the research on the application of temperature-sensitive tackifiers in oil and gas extraction mainly focuses on three aspects, pre-extraction preparation, water injection extraction, and post-extraction wastewater treatment. With the advancement of science and technology and the increasing demand for high-performance drilling fluids, research and the application of temperature-sensitive tackifiers will develop in the following directions: improving sensitivity and stability, continuing to optimize the molecular structure of temperature-sensitive tackifiers to improve their sensitivity to temperature changes and stability under extreme conditions.

## 4. Conclusions

In the fields of oil and gas drilling and production, temperature-sensitive materials have attracted increasing attention from scholars due to their outstanding performance and broad application prospects. This article focuses on the primary applications of temperature-sensitive materials in oil and gas drilling and draws the following conclusions:

1. With regard to temperature-sensitive plugging agents, they have shown significant effects in plugging formation fractures and reducing drilling fluid losses. However, in the face of complex underground environments and changeable operating conditions, especially in multi-well coordinated operations and high-temperature and high-pressure environments, the performance of plugging agents still needs to be further improved.
2. Regarding thickening agents and enhanced oil recovery agents, they have tremendous potential in increasing oil field recovery rates due to their excellent temperature-sensitive properties. These materials offer extensive application prospects. The authors of this article believe that future research directions will mainly focus on areas such as copolymerization and graft modification to further develop thickening agents and enhanced oil recovery agents.
3. In the treatment of waste drilling fluid, temperature-sensitive materials are mainly used as flocculants, and their temperature-responsive hydrophilic and hydrophobic changes are used to effectively separate oil and solid particles. However, the sensitivity of temperature response and the precise control of low critical solution temperature (LCST) are still challenges. Therefore, future research needs to pay more attention to the molecular design and application optimization of these materials in order to better adapt to the wastewater treatment needs of different oil fields.

In the past hundred years since the advent of temperature-sensitive materials, due to their unique properties and wide application prospects in various fields, they have aroused the interest of more and more researchers. With the continuous progress of polymer science, its synthesis methods have also been significantly improved. Although temperature-sensitive materials have shown great potential in oil and gas drilling, there are still many challenges, including their long-term stability, economic cost, and adaptability to complex geological conditions. Future research should focus on optimizing the properties of these materials and exploring new synthesis and modification methods. In addition, with the continuous advancement of green chemistry and sustainable development concepts, the development of new temperature-sensitive materials will also become an important direction for future research.

## Figures and Tables

**Table 1:** Advantages and disadvantages of three temperature-sensitive polymer materials.

	Advantages	Disadvantages	Applications
N-substituted acrylamide polymers	Temperature-responsive and very adjustable	Biocompatibility and higher preparation costs in some cases	Water-driven oil recovery agents, water-based drilling fluids
Amphiphilic block copolymers	Modulation of material properties during phase transitions, biocompatible	Complex synthetic processes and toxicity of some copolymers	Reservoir improvement, well plugging agents
Peptides	Natural origin, high biocompatibility, good adjustability	Difficult synthesis, stability issues, and high cost	Water-based lubricants, smart release in reservoirs

## Data Availability

Data sharing is not applicable to this article as no new data were created or analyzed in this study.
